# Comparison of Monovalent and Divalent Ions Removal from Aqueous Solutions Using Agricultural Waste Biochars Prepared at Different Temperatures—Experimental and Model Study

**DOI:** 10.3390/ijms21165851

**Published:** 2020-08-14

**Authors:** Agnieszka Tomczyk, Zofia Sokołowska, Patrycja Boguta, Katarzyna Szewczuk-Karpisz

**Affiliations:** Institute of Agrophysics, Polish Academy of Sciences, Doświadczalna 4, 20-290 Lublin, Poland; z.sokolowska@ipan.lublin.pl (Z.S.); p.warchulska@ipan.lublin.pl (P.B.); k.szewczuk-karpisz@ipan.lublin.pl (K.S.-K.)

**Keywords:** adsorption mechanism, Ag and Cu, agricultural biochar, Langmuir-Freundlich model

## Abstract

Copper (Cu) and silver (Ag) occur naturally in the environment but have toxic effects on organisms at elevated concentrations. This paper discussed the removal of Cu and Ag from aqueous solutions using biochars obtained at different pyrolysis temperatures. Three biomass sources—sunflower husks (SH), a mixture of sunflower husks and rapeseed pomace (SR) and wood waste (WW)—were pyrolyzed at 300, 400 and 500 °C. Biochars produced at 500 °C exhibited a higher specific surface area, lower variable surface charge and lower contents of surface functional groups than those obtained at 400 or 300 °C. The pseudo-second-order model and intra-particle diffusion (IPD) model well-described the Cu and Ag adsorption kinetics. The Cu adsorption was about 1.48 times slower than the Ag adsorption on the biochars obtained at 500 °C. The model of Langmuir-Freundlich well-described the equilibrium adsorption. Agricultural biochars obtained at >500 °C had a surface with a higher affinity to attract Ag than Cu and were able to remove a larger amount of heavy metals from aqueous media than those prepared at lower pyrolysis temperatures.

## 1. Introduction

Silver (Ag) is classified as a voluntarily enforced secondary contaminant [1]. Silver, which occurs in nature as a mineral (argentite or chlorargyrite) [2], has a wide spectrum of applications, including jewelry; clothing; dentistry; cosmetics; fertilizers and bacterial-fungal, mycobacterial and virucidal agents [3]. These varied uses of Ag can easily increase its concentration in wastewater and groundwater. The drinking water guideline for silver in drinking water is >0.05 mg/L and, in wastewater, is >0.1 mg/L. In turn, the average silver content of the effluent water is 50 µg/L. Excessive accumulation of Ag in the body can cause a disease called “argyria” [3]. Copper (Cu) occurs as a natural mineral (azurite and malachite), heavy metal and microelement. The EPA (United States Environmental Protection Agency) announced that the maximum contaminant level in aquatic environments for copper is 1.3 mg/L [4]. Cu is used in fertilizers, electronics, pharmaceuticals, dyes and textiles, coal combustion and the production of plant protection products [5]. Copper participates in the photosynthesis and respiration of organisms at relatively low concentrations [6]. Excess Cu accumulates in the roots and leaves of plants and causes many metabolic disorders, such as the limitation of growth and development, DNA damage and reduction of photosynthesis processes [6]. A high concentration of Cu evokes changes in the nasal mucosa, gastritis, diarrhea and toxicity symptoms such as chronic lung damage in the human body [6].

Several technologies, methods and processes of heavy metal pollution removal from aqueous media were exploited, e.g., chemical precipitation, filtration, osmosis, chemical oxidation and many others. These chemical and physical processes become expensive and inefficient when the concentration of a contamination is too low. Thus, there is a need to develop an efficient method of removing Ag and Cu from aqueous media that is not only cheap and effective but, also, eco-friendly to the environment. Many researchers have demonstrated that the adsorption process is the most suitable for protects of aqueous media among the various potential technologies. This technique is one of the most efficient methods for heavy metal removal, characterized by low energy consumption, low cost, high effectiveness, high eco-friendliness and ease of operation [7]. Adsorption is a surface process, where adsorbates (molecules, ions and atoms) adhere to the substrate by chemical or physical interactions [8]. Various adsorbents (natural and synthetic) for Cu and Ag removal have been investigated, including minerals, activated carbon, biochar, composite materials [9,10,11,12,13,14,15,16,17,18,19], nanosized iron oxides, magnesium oxides manganese oxides, aluminum oxides, titanium oxides and cerium oxides. However, sorbents of carbon origin (e.g., activated carbon and biochar) have exhibited the most benefits in removing organic, as well as inorganic, impurities [20]. Thus, much of the recent work has focused on biochar as an inexpensive, economical and eco-friendly adsorbent.

Biochar is a carbonaceous material that exhibits negligible toxicity, a high content of organic carbon and optimal concentrations of micro- and macroelements (potassium, sodium, magnesium, calcium, etc.) [21]. Biochars removal heavy metals by the process of adsorption, which is important for environmental protection and management [22,23]. Biochars have two different fractions: a carbonized and a noncarbonized. They interact with water contaminants through oxygen surface functional groups: carboxyl, phenolic, hydroxyl and lactonic [24]. These fractions play main roles in the adsorption process [25]. The physicochemical properties of biochar, the molecular structure and pore size distribution are dependent on the type of biomass and pyrolysis conditions, particularly the value of pyrolysis temperature [26]. It affects the biochar adsorption capacity [20].

Adsorption capacities of low- and high-temperature biochars as Cu and Ag adsorbents were investigated. In other words, a comprehensive research on the pyrolysis temperature effects on the biochar adsorption efficiency relative to monovalent and divalent metal ions was performed. The experimental results provided that the metal valence affected its adsorption on the biochar surface, which has not yet been reported in the literature. The experimental research investigation of the obtained data provided three main objectives: (i) investigate the effects of the pyrolysis temperature on the surface characteristics of biochar, (ii) study the kinetics and equilibrium isotherms of the adsorption of Cu and Ag onto biochar produced at various pyrolysis temperatures and (iii) evaluate the biochars’ efficiency at the removal of monovalent and divalent metals from aqueous media. The research demonstrated that the adsorption on biochars will be helpful and important as practical applications, especially for the removal of metal ions from wastewater using natural adsorbents.

## 2. Results and Discussion

### 2.1. Effect of Pyrolysis Temperature on Biochar Surface Properties

**Figure 1:**
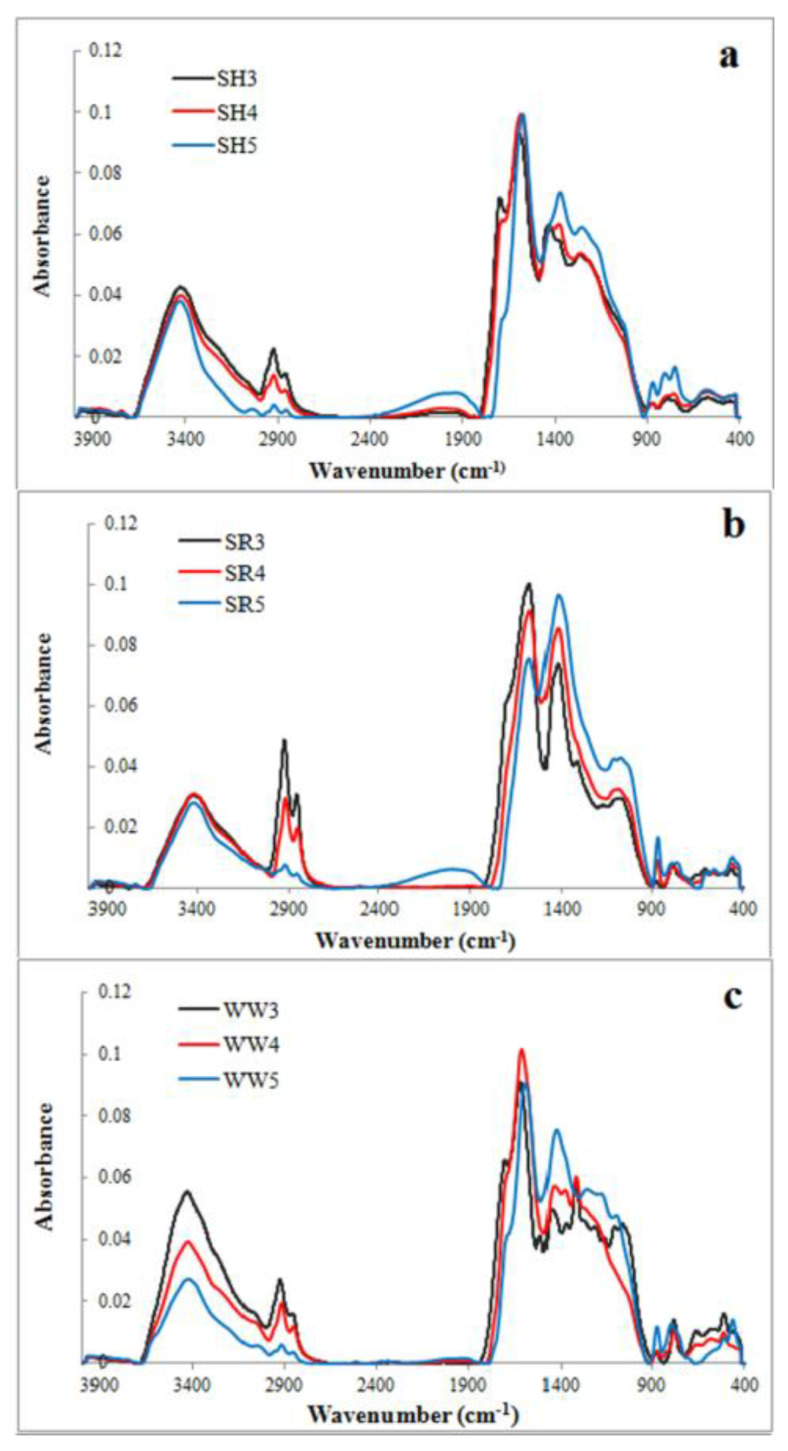
FTIR spectra of experimental biochars (**a**) biochar derived from sunflower husks; (**b**) biochar derived from sunflower husks and rapeseed pomace; (**c**) biochar derived from wood waste): SH3 (sunflower husks, 300 °C), SH4 (sunflower husks, 400 °C), SH5 (sunflower husks, 500 °C), SR3 (sunflower husks + rapeseed pomace, 300 °C), SR4 (sunflower husks + rapeseed pomace, 400 °C), SR5 (sunflower husks + rapeseed pomace, 500 °C), WW3 (wood waste, 300 °C), WW4 (wood waste, 400 °C) and WW5 (wood waste, 500 °C).

Characteristic surface results of the all experimental agricultural biochars are demonstrated in Table 1, and Figure 1a–c provided the differences between the natural adsorbents obtained at various pyrolysis temperatures.

The examined biochars were characterized by a well-developed specific surface area (*S_BET_* above 53.1 m^2^/g) and basic pH (pH > 8). The specific surface area and pH of the experimental biochars increased with the increasing value of the pyrolysis temperature. This is caused by the decomposition of organic matter and the destruction of aliphatic alkyls and ester groups, as well as the aromatic lignin or cellulose core [27]. Sun et al. The authors of [28] also observed that biochars (bagasse-derived) obtained at 300 °C had a lower *S_BET_* value (5.2 m^2^/g) than biochars obtained at 450 °C (13.6 m^2^/g).

Biochars exhibited a lower value of *Q* when the pyrolysis temperature was higher. The negative charge of active sites on the experimental biochar surface, which is responsible for the value of *Q*, is attributed to surface groups -COOH and -OH [27]. The natural adsorbent surface oxygen functional group’s removal was caused by a higher value of the pyrolysis temperature. This was indicated by the contents of the surface acidic functional groups determined by Boehm’s titration method and the O/C ratio determined by elemental analysis. The lower contents of the functional groups are due to dehydration, deoxygenation and decarboxylation of the biomass [24,29]. Sun et al. [28] observed that the ratio O/C of biochar decreased with the increasing pyrolysis temperature.

The H/C ratio of the examined biochars also decreased with the increasing pyrolysis temperature, which was attributed to increasing the hydrophobic character of the biochar. Lignins, which are in biochar biomasses, are not converted into hydrophobic polycyclic aromatic hydrocarbons at temperatures lower than 500 °C [30]. Jindo et al. [31] reported that the H/C ratio of biochars derived from agricultural residues and obtained at 400, 500, 600, 700 and 800 °C decreased with the increasing pyrolysis temperature. Schimmelpfennig et al. [32] demonstrated that both H/C and O/C decrease with the increasing pyrolysis temperature.

FTIR spectra of the nine evaluated biochars are illustrated in Figure 1a–c. These spectra reflect changes in the surface functional groups of biochars obtained at different temperatures. The bands observed in the spectra represent the following: the -OH groups stretching (~3500 cm^−1^), methyl C-H stretching (~2930 cm^−1^), methylene C-H stretching (~2860 cm^−1^), aromatic C=C and the C=O stretching of conjugated ketones and quinones (~1600 cm^−1^), C=C stretching of the aromatic structures (~1430 cm^−1^) and C-O-C stretching of the aryl ethers, as well as the phenolic compounds associated with the degradation of lignin (~1238 cm^−1^). C-O-C and –OH stretching in the ester groups was associated with a cellulose and hemicellulose degradation of ~1130 cm^−1^ and aromatic C-H (~815 cm^−1^). All of these FTIR spectra bands are typical for biochars [33].

The band intensity at 3500 cm^−1^, corresponding with the -OH groups, decreased dramatically for the wood waste (WW) biochars and slightly for the sunflower husk (SH) and sunflower husk + rapeseed pomace (SR) biochars with the increasing temperature. The bands attributed to methyl, methylene and aromatic stretching decreased with the increasing pyrolysis temperature, but the intensities of the bands at 1430 cm^−1^ and 815 cm^−1^ increased. The band at 1138 cm^−1^ was characteristic for all SR biochars and for wood waste 300 °C (WW3). For the WW and SH biochars, there was also a visible band at 1238 cm^−1^, which was attributed to the surface functional phenolic -OH groups created by cellulose and hemicelluloses degradation [33]. A lower magnitude of the bands suggested a decrease in the polar surface oxygen acidic functional groups when increasing the values of the pyrolysis temperature [34]. Biochars that produced >500 °C exhibited the greatest loss of functional groups and higher aromaticity/hydrophobicity.

### 2.2. Adsorption of Monovalent and Divalent Metals on Biochar—Kinetics Studies

Kinetics model parameters were obtained with the linear fitting procedure (Equations (2)–(4)) and inserted in Table 2 and Table 3. The pseudo-second-order model described the adsorption data better for all the experimental biochars. Correlation coefficients for the pseudo-first-order model ranged from 0.81 to 0.89 for both metals, whereas, for the pseudo-second-order model, ranged from 0.98 to 0.99 (higher values of the correlation coefficients (*R*^2^) indicated that the selected model described the kinetics data well). A good fit to the pseudo-second-order model suggested that the chemisorption process depended predominantly on the valence and covalent forces and the surface charges of the biochars [35,36].

The rate constants of the Cu or Ag adsorption process on the experimental biochars increased with the increasing temperature. The *k*_2_ values of the Cu adsorption increased in the range 3.5–11.0 × 10^−2^ g/mg·min for SR, 4.5–8.2 × 10^−2^ for SH and 1.0–1.9 × 10^−2^ for WW. The *k*_2_ values of the Ag adsorption increased in the range 5.1–8.3 × 10^−2^ g/mg·min for SR, 4.2–6.1 × 10^−2^ for SH and 1.5–2.2 × 10^−2^ for WW. The fastest adsorption occurred on the biochars obtained at 500 °C, but the Cu adsorption was about 1.48 times slower than the Ag adsorption. Furthermore, the values of the experimental *q_e_* and calculated biochars kinetics increased with the increasing the values of the pyrolysis temperature and were very similar. The experimental *q_e_* values of the Cu adsorption increased in the range 9.0–9.6 mg/g for SR, 9.0–9.6 for SH and 6.3–7.2 for WW. The experimental *q_e_* values of the Ag adsorption increased in the range 9.1–10.0 g/mg·min for SR, 9.9–10.0 for SH and 9.2–10.0 for WW. The calculated *q_e_* values of the Cu adsorption increased in the range 9.0–9.9 mg/g for SR, 9.0–9.6 for SH and 6.3–7.2 for WW. The calculated *q_e_* values of the Ag adsorption increased in the range 9.1–10.1 g/mg·min for SR, 9.9–10.0 for SH and 9.2–10.0 for WW. Kołodyńska et al. [36] reported that the *k*_2_ and *q_e_* values for Cu removal on biochars obtained from animal manure (pig and cow) at 400 and 600 °C increased with increasing the values of the pyrolysis temperature (*k*_2_: 0.1 and 0.8 g/mg·min and *q_e_*: 5.9 and 6.2 mg/g).

**Figure 2:**
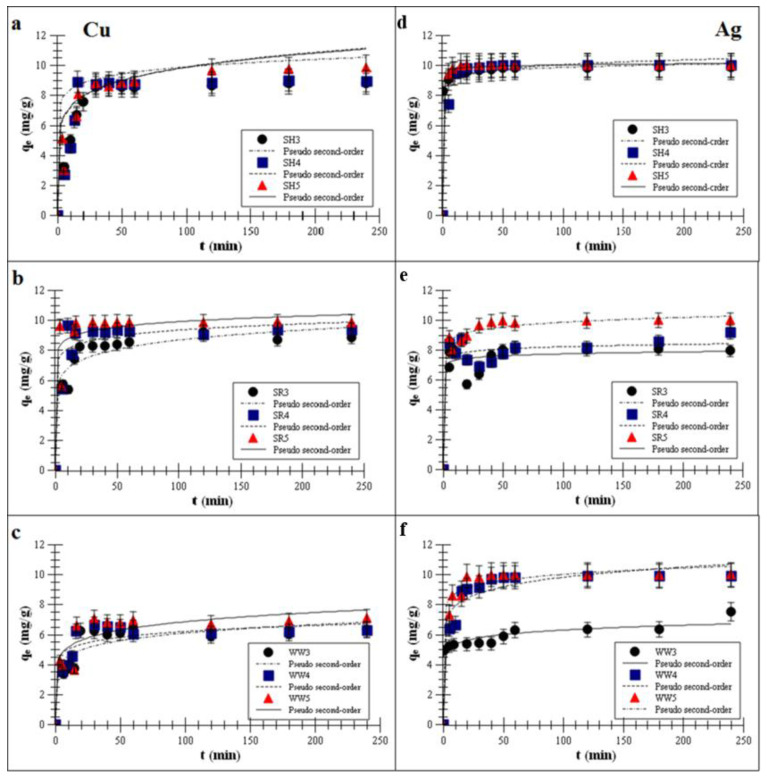
Pseudo-second-order fitting [35,36] of the metal adsorptions on the biochars (**a**,**d**)biochar derived from sunflower husks; (**b**,**e**) biochar derived from sunflower husks and rapeseed pomace; (**c**,**f**) biochar derived from wood waste): SH3 (sunflower husks, 300 °C), SH4 (sunflower husks, 400 °C), SH5 (sunflower husks, 500 °C), SR3 (sunflower husks + rapeseed pomace, 300 °C), SR4 (sunflower husks + rapeseed pomace, 400 °C), SR5 (sunflower husks + rapeseed pomace, 500 °C), WW3 (wood waste, 300 °C), WW4 (wood waste, 400 °C) and WW5 (wood waste, 500 °C); *q_e_* is the heavy metal removal capacity at equilibrium; *t* is the time; Cu is copper and Ag is silver.

Plots of the pseudo-second-order fitting are shown in Figure 2a–f.

The kinetics data showed that the Ag and Cu adsorption process on all experimental biochars reached equilibrium in less than one h. Such a short time suggests strong interactions between the active sites on biochar surfaces and Cu or Ag ions. Once the isotherm reached a plateau, there was no change in the adsorption over time. Reaching of the plateau was faster for the biochars produced at 500 °C than for those produced at lower pyrolysis temperatures, and it took approx. 40–50 min to reach a total adsorption efficiency for Cu of approx. 99.3% ± 15.7%. For Ag, reaching a plateau took approx. 30–40 min (99.9% ± 3.9%). Mohan et al. [37] reported that divalent ions were adsorbed at the level of 40–70% ± 18.7% on wood-derived biochars within about one h. Cibati et al. [38] showed that the adsorption of Cu on biochars can achieve an adsorption equilibrium in 60 min. Wang et al. [39] reported that the adsorption kinetics of monovalent ions were quite fast in the first h, and Jeon [40] further showed that Ag removal using waste coffee grounds achieved equilibrium in less than one h. Awual [9] observed that the maximum Cu adsorption percentage on composite materials was attained after 60 min of stirring time. One hour was sufficient to achieve an equilibrium adsorption for Cu by various materials.

**Figure 3:**
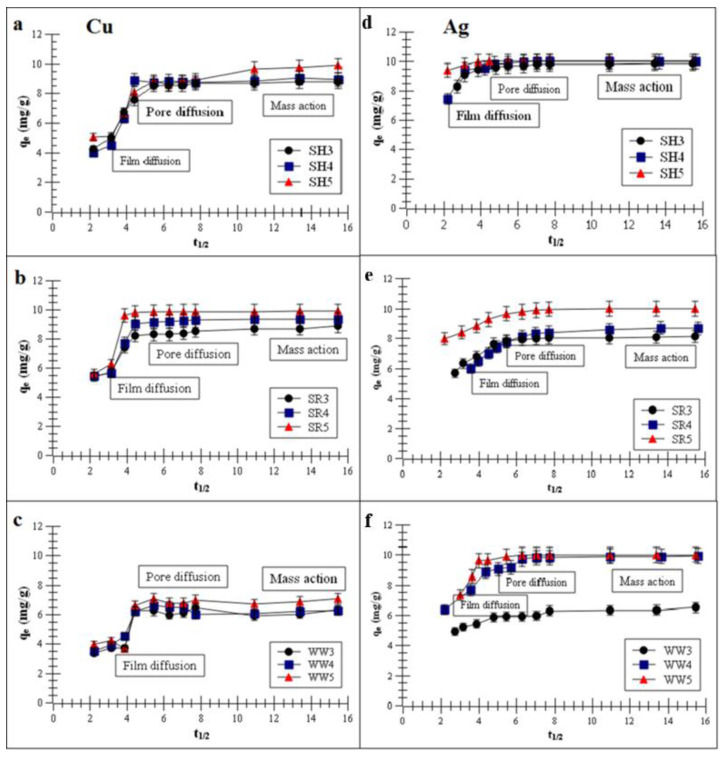
Intra-particle diffusion (IPD) model of the metal adsorptions on the biochars (**a**,**d**) biochar derived from sunflower husks; (**b**,**e**) biochar derived from sunflower husks and rapeseed pomace; (**c**,**f**) biochar derived from wood waste): SH3 (sunflower husks, 300 °C), SH4 (sunflower husks, 400 °C), SH5 (sunflower husks, 500 °C), SR3 (sunflower husks + rapeseed pomace, 300 °C), SR4 (sunflower husks + rapeseed pomace, 400 °C), SR5 (sunflower husks + rapeseed pomace, 500 °C), WW3 (wood waste, 300 °C), WW4 (wood waste, 400 °C) and WW5 (wood waste, 500 °C); *q_e_* is the heavy metal removal capacity at equilibrium and *t*_1/2_ is half-adsorption time.

Figure 3a–f presents the plots of the metal removal capacity vs. *t*^1/2^ (half-adsorption time).

The intra-particle diffusion (IPD) model, which described the experimental data, suggests that Cu and Ag adsorption had three stages: film diffusion, pore diffusion and mass action. The first stage presented film diffusion, the second stage was characterized by the Cu or Ag adsorption process on the biochar inner surfaces of macropores and the third stage was characterized by the metals adsorption process in the capillary spaces of the biochars [37,41].

### 2.3. Adsorption of Monovalent and Divalent Metals on Biochar—Equilibrium Isotherm Studies

The adsorption data of the Cu or Ag adsorption process on the experimental biochars were analyzed using three models: the Freundlich, Langmuir and Langmuir-Freundlich models. The calculated isotherm parameters of the Cu and Ag adsorptions (Equations (5)–(7)) were summarized in Table 4 and Table 5.

The correlation coefficient values of all the experimental biochars were: <0.90 for the Freundlich model, <0.95 for the Langmuir model and >0.98 for the Langmuir-Freundlich model. Data for the adsorption of the above-mentioned metals on all biochars fitted most closely to the Langmuir-Freundlich (*R^2^* > 0.98) model.

The Langmuir-Freundlich monolayer capacity, *A_m_*, increased with the increasing pyrolysis temperature. This indicates that the biochar obtained at a higher pyrolysis temperature (>500 °C) had a better affinity for Cu and Ag adsorptions. This is confirmed by the increasing *K_LF_* constant (surface affinity for adsorbate adsorption) and decreasing parameter *m* (surface heterogeneity). Furthermore, the obtained parameters show that the biochar had a greater adsorption affinity for Ag than for Cu. These results indicate that the biochar that produced >500 °C exhibited the highest surface affinity for metal ions. This was consistent with the higher value of the specific surface area (*S_BET_*). Kołodyńska et al. [36] observed that the *S_BET_* of biochars derived from pig and cow manure obtained at 400 and 600 °C also increased with the increasing temperatures (15.6 to 15.9 m^2^/g and 2.5 to 8.0 m^2^/g, respectively). An increased pyrolysis temperature contributed to the removal of tar particles (ketones, aldehydes, organic liquids and PAHs—polycyclic aromatic hydrocarbons) from the micropores, which ultimately increased the total pore volume and *S_BET_* value [31,42]. The adsorbate entered the biochar pores during the adsorption process, and the close proximity of carbon atoms within the mezzo- and micropores resulted in the retention of Cu and Ag by the van der Waals forces [43]. Additionally, the phenomenon of surface precipitation was also possible [37,44]. This was another mechanism accountable for the immobilization of heavy metal contaminants through biochar. It involved the formation of solids with PO_4_^3−^, CO_3_^2−^, Cl^−^ or SO_4_^2−^ either on the surface or in the solution during the adsorption process. Zhou et al. [45] reported the surface precipitations for divalent metals on biochar obtained at 400 °C.

Between the surfaces of the biochar and cations (Cu, Zn, Pb and Ag), there was an electrostatic attraction during the heavy metal adsorption process [46]. The variable surface charge (*Q*) measured for the used solids came mainly from negative surface groups. However, higher pyrolysis temperatures were observed to be associated with lower *Q* values. A high carbonization temperature (above 400 °C) gave biochars a graphene structure and contributed to the removal of the surface functional groups [47]. As a result, the electrostatic attraction between the heavy metals and adsorbent was weakened [48,49]. However, the higher Cu and Ag adsorption levels noted for the biochars prepared at higher temperatures proved that electrostatic interactions were not the main forces determining the adsorption process.

**Figure 4:**
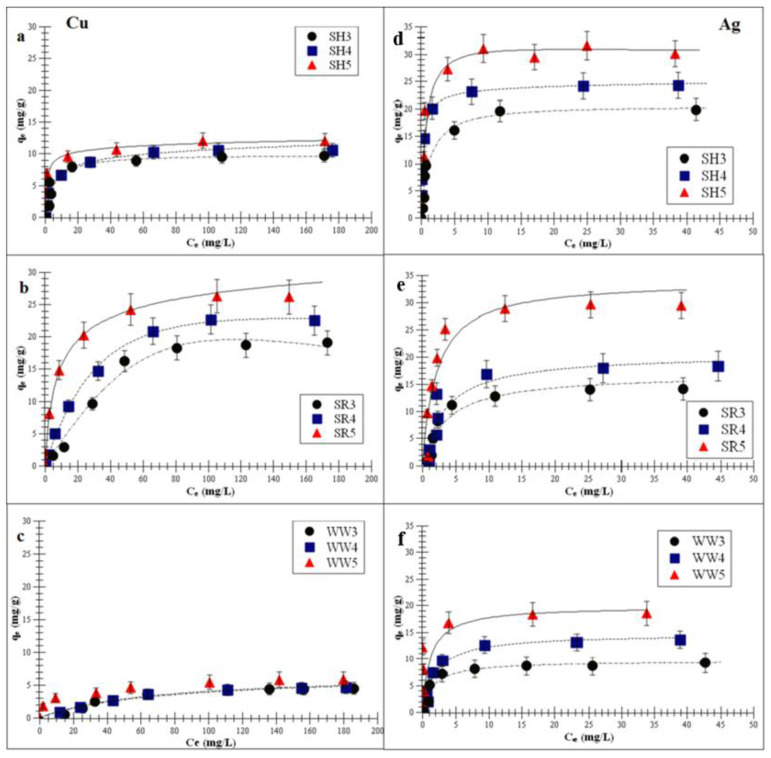
Langmuir-Freundlich isotherm fitting of Cu and Ag adsorptions on the experimental biochar (**a**,**d**) biochar derived from sunflower husks; (**b**,**e**) biochar derived from sunflower husks and rapeseed pomace; (**c**,**f**) biochar derived from wood waste): SH3 (sunflower husks, 300 °C), SH4 (sunflower husks, 400 °C), SH5 (sunflower husks, 500 °C), SR3 (sunflower husks + rapeseed pomace, 300 °C), SR4 (sunflower husks + rapeseed pomace, 400 °C), SR5 (sunflower husks + rapeseed pomace, 500 °C), WW3 (wood waste, 300 °C), WW4 (wood waste, 400 °C) and WW5 (wood waste, 500 °C); *q_e_* is the amount of adsorbed metal ions at equilibrium and *C_e_* is the equilibrium concentration of ions in the solution.

Figure 4a–f demonstrates the Langmuir-Freundlich isotherm fitting of Cu or Ag adsorptions on the experimental biochars.

**Figure 5:**
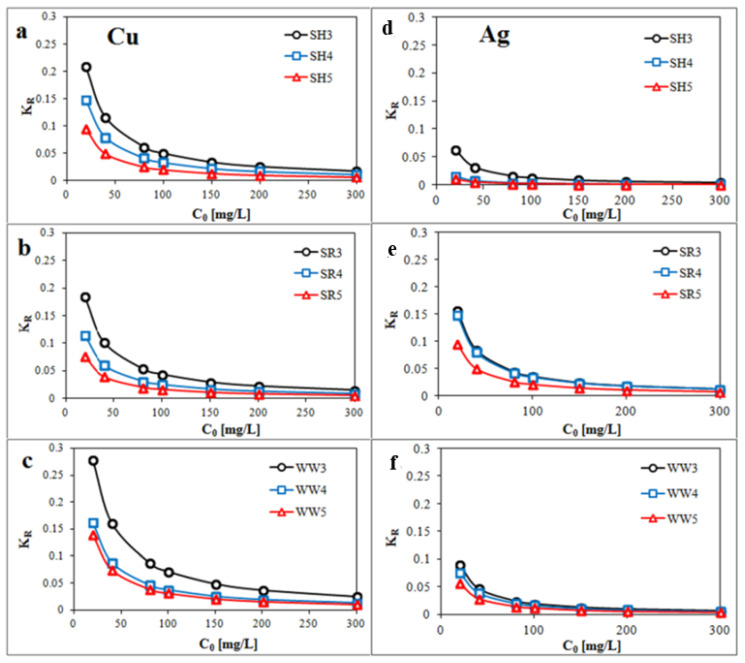
Plot of *K_R_* (dimensionless constant separation factor) against the initial concentration *C_o_* (**a**,**d**) biochar derived from sunflower husks; (**b**,**e**) biochar derived from sunflower husks and rapeseed pomace; (**c**,**f**) biochar derived from wood waste): SH3 (sunflower husks, 300 °C), SH4 (sunflower husks, 400 °C), SH5 (sunflower husks, 500 °C), SR3 (sunflower husks + rapeseed pomace, 300 °C), SR4 (sunflower husks + rapeseed pomace, 400 °C), SR5 (sunflower husks + rapeseed pomace, 500 °C), WW3 (wood waste, 300 °C), WW4 (wood waste, 400 °C) and WW5 (wood waste, 500 °C).

Parameter *K_R_* (Figure 5a–f) was calculated by Equation (8) and showed that the Cu and Ag removals by the all experimental biochars were favorable.

Specific surface areas of the biochars occupied by Cu and Ag, calculated from Equation (9), are summarized in Table 6.

Specific surface areas occupied by Cu or Ag on the biochars increased with the increasing pyrolysis temperature. This phenomenon was associated with the different *S_BET_* values of the biochars, the ionic radius of the adsorbates (Cu = 0.72 Å and Ag = 1.13 Å) and their electronegativity (Cu = 1.90 and Ag = 1.93). The values of specific surface area occupied by Ag on the experimental biochars were higher than that occupied by Cu. This was caused by the stronger attraction between the surface functional groups and Ag ions, which facilitated their penetration into the biochar pores. Cations can be attracted by oxygen (O), which is a component of the -COOH, -C=O and -OH groups of the biochars obtained at lower temperatures [50]. These surface functional groups containing mono- or multi-donor atoms may form coordination bonds with adsorbates (Lewis bases or Lewis acids) [51]. Depending on the amount of ligand donor atoms, biochar surface functional groups are called mono-, double- or polydentates [52]. Cu can form a bond with two oxygen atoms of adjacent carboxyl groups, while Ag can do so with one carboxyl group. An ion exchange is also possible. This process involves the exchange of ionized cations and protons on the biochar surfaces with dissolved metals [46] and depends on the surface functional groups and metal ionic radius. The ion exchanges between ions present above the biochar surfaces (Na^+^, K^+^ or Ca^2+^ and Mg^2+^) and ions in the solution [53] were observed in many cases.

The removal efficiency of the all the experimental biochars, calculated from Equation (10), is summarized in Table 7.

These results also show that all the experimental biochar efficiencies increased with the increasing pyrolysis temperature, and Cu was adsorbed with a lower efficiency than Ag. This additionally proved that the biochar obtained at the highest temperature, characterized by the largest specific surface area, possessed the highest affinity for the adsorption of heavy metal ions. However, it had a larger adsorption capacity for monovalent ions than for divalent ones.

The obtained experimental results were compared with the biochar efficiencies for metal adsorption reported by other scientists. Komkiene and Baltrenaite [54] proved that the removal efficiency for heavy metal ions was approx. 35–37% on silver birch-derived biochar, and the differences in biochar efficiency were associated with various temperatures of the pyrolysis. Chen et al. [55] reported that corn straw biochar (produced at 600 °C) and wood biochar (produced at 450 °C) had high heavy metal adsorption efficiencies, and the Cu removal efficiency was 56.7% for wood biochar and 98.3% for corn straw biochar. Zhou et al. [56] reported that bamboo biochar removed approx. 37%, and zerovalent iron-biochar composites removed 100%, of Ag.

## 3. Materials and Methods

### 3.1. Biochar Production

Biochars were derived from three source materials: sunflower husks (SH), a mixture of 50% sunflower husks and 50% rapeseed pomace (SR) and wood waste (WW). Biochars were produced by the Czestochowa University of Technology via a 30-min process of autothermic biomass pyrolysis at 300, 400 and 500 °C under oxygen-limited conditions. Processes of autothermic pyrolysis were performed in the reactor under pressure and flow conditions that would ensure the maximum rate of heating of the fragmented biomass. More details of the biochar preparation are given elsewhere [57,58,59,60,61]. Pyrolysis products were air-dried, crushed and sieved through a 2-mm mesh. Biochar samples were denoted as SH3 (sunflower husks, 300 °C), SH4 (sunflower husks, 400 °C), SH5 (sunflower husks, 500 °C), SR3 (sunflower husks + rapeseed pomace, 300 °C), SR4 (sunflower husks + rapeseed pomace, 400 °C), SR5 (sunflower husks + rapeseed pomace, 500 °C), WW3 (wood waste, 300 °C), WW4 (wood waste, 400 °C) and WW5 (wood waste, 500 °C), respectively.

### 3.2. Surface Characteristics of Biochars

Natural organic adsorbent surface characteristics were investigated by various conventional physicochemical methods. Variable surface charge was established by potentiometric titration with Titrino 702 SM (Metrohm, Herisau, Switzerland) [62]. Contents of surface functional groups, such as lactonic, carboxylic and phenolic, were determined by the Boehm titration method [63]. This method is based on the assumption that the acidic constants of carboxylic, lactonic and phenolic groups differ by several orders of magnitude, and therefore, it is possible to neutralize these groups with properly selected reagents. The acidity of individual functional groups depends on their location and environment, i.e., on the size of the layers and the type or position of the other substituent. The pH value was measured electrochemically, the ratio 1:10 was used and the pH was determined after 1 h of equilibration using a digital pH meter (Multifunction pH-meter CX-505, Elmetron, Zabrze, Poland) [64]. Biochar specific surface areas were measured from the water vapor adsorption/desorption isotherm method according to the Polish standard [65]. H/C and O/C ratios were analyzed using an elemental analyzer (2400 CHNS/O Analyzer Series II, PerkinElmer, Waltham, MA, USA). FTIR spectrometer (Tensor 27, Bruker, Billerica, MA, USA) provided Fourier-transform infrared (FTIR) spectra of biochars. One milligram of biochars were homogenized with 200 mg KBr of spectral purity and analyzed in the range of 400–4000 cm^−1^. The characteristics were obtained as an average of three measurements, with 256 scans at 2-cm^−1^ resolutions each.

### 3.3. Adsorption Study for Aqueous Systems with Monovalent and Divalent Metals

Metal solutions were prepared by dissolving appropriate amounts of Cu(NO_3_)_2_∙2H_2_O (Sigma Aldrich, St. Louis, MA, USA) or anhydrous AgNO_3_ (Sigma Aldrich, St. Louis, MA, USA) in distilled water to obtain final metal concentrations of 0–300 mg/L. Adsorption was conducted in a batch experiment at room temperature (20 ± 2 °C). Biochar (0.15 g) was added to 15 mL of Cu or Ag solution (for kinetics: 100 mg/L and for equilibrium adsorption: 0–300 mg/L) and shaken using a laboratory rotator (for kinetics: 5–240 min and for adsorption: 60 min). Optimization conditions of Cu or Ag adsorption process on biochars and ratio 1:100 were chosen based on our previous research and the latest literature [66,67,68]. By using 0.10M HNO_3_ (nitric acid purum ≥65%, Sigma Aldrich, St. Louis, MA, USA), the system pH (Ag or Cu/biochar) was adjusted to 5.0, and this value was chosen to avoid metal precipitation. At pH < 6, the concentrations of metal hydroxide forms such as MOH^+^, M(OH)_2_, M(OH)_3_^−^ and M(OH)_4_^2−^ are insignificant and do not affect the sorption process of Ag^+^ or Cu^2+^ ions [39]. After shaking, the solutions were filtered, and the concentrations of Cu or Ag were analyzed by AAS (atomic absorption spectrometry, contra 300, Analityk Jena, Jena, Germany). AAS uses an oxyacetylene flame. Cu was detected at a wavelength of 325 nm and calibration curve in the range of 0–5 ppm. Ag was detected at a wavelength of 328 nm and a calibration curve in the range of 0–1 ppm. The samples were diluted before the measurements started.

Batch experiment of the adsorption process on biochars and surface properties measurements were performed in triplicate, and the points of the graphs were obtained from the averaged data.

Cu and Ag adsorption amounts on experimental biochars, *q_e_*, can be calculated as follows:

(1) $$q_{e}\;=\;\frac{\left(C_{0}\;-\;Ce\right)\cdot V}{m}$$

where *q_e_* is the adsorption amount of Cu or Ag (mg/g), *C*_0_ is the initial concentration of heavy metal (mg/L), *C_e_* is the concentration of heavy metal at equilibrium (mg/L), *m* is the mass of the sample (mg) and *V* is the volume of the solution (L).

Kinetics of Cu or Ag adsorption on experimental biochars can be described by the pseudo-second-order [69,70] (Equation (3)) and pseudo-first-order models [71] (Equation (2)):

(2) $$\ln\left(q_{e}-q_{t}\right)=\ln q_{e}-k_{1}\cdot t$$

(3) $$\frac{t}{q_{t}}=\frac{1}{k_{2}\cdot q_{e}^{2}}+\frac{t}{q_{e}}$$

where *q_t_* (mg/g) is the heavy metal removal capacity at time *t* (min), *q_e_* (mg/g) is the heavy metal removal capacity at equilibrium and *k*_1_ (1/min) and *k*_2_ (g/mg·min) are the reaction rate constants.

Kinetic Cu and Ag adsorption data can be described using the intra-particle diffusion (IPD) model [72]:

(4) $$q_{t}=k_{D}\cdot t^{\frac{1}{2}}+C$$

where *k_D_* (g/mg·min^1/2^) is the IPD rate constant, and *C* (mg/g) is a constant. If the plot of *q_t_* vs. *t*^1/2^ exhibits multilinearity, then each linear segment can be attributed to a different physical mechanism.

Equilibrium adsorption of Cu or Ag on experimental biochars was described by the Freundlich (Equation (5)), Langmuir (Equation (6)) and Langmuir-Freundlich (Equation (7)) isotherms.

Freundlich isotherm of equilibrium Cu and Ag adsorptions on biochars is expressed as follows:

(5) $$q_{e}=K_{F}\;{\left[\frac{C_{e}}{\;a_{m}}\right]}^{n}$$

where *q_e_* is the amount of adsorbed metal ions at equilibrium (mg/g), *C_e_* is the equilibrium concentration of ions in the solution (mg/L) and *K_F_* (in units of *q_e_*) and *n*, 0 < *n* < 1, are the Freundlich constants (mg/g(L/mg)^1/*n*^), which represent the sorption capacity and the “heterogeneity parameter” [73], respectively.

Langmuir isotherm [74] has the following expression:

(6) $$q_{e}=\frac{Q_{m}K_{L}C_{e}}{1+K_{L}C_{e}}$$

where *Q_m_* is the maximum amount of metal ions in the monomolecular layer (mg/g), and *K_L_* is the Langmuir constant related to the affinity of the adsorbate for active sites (L/mg).

Langmuir-Freundlich equation describes monolayer adsorption on an energetically heterogeneous surface with a quasi-Gaussian energy distribution function. It has the following form [75]:

(7) $$\frac{q_{e}}{A_{m}}={\left\{\frac{{\left(K_{LF}C_{e}\right)}^{m}}{\left[1+{\left(K_{LF}C_{e}\right)}^{m}\right]}\right\}}^{\frac{n}{m}}$$

where the constant of the Langmuir-Freundlich equation, *K_LF_*, is related to the affinity of the adsorbate for active sites (L/mg), *A_m_* is the amount of available surface sites (mg/g) and *n* and *m* (0 < *n*, *m* ≤ 1) are the parameters determining the shape of the energy distribution function.

Effect of isotherm shape can be used to predict whether a given sorption system is “favorable” or “unfavorable” in batch processes [75]. Dimensionless constant separation factor (*K_R_*) of Cu and Ag adsorption can be expressed as

(8) $$K_{R}=\frac{1}{1+K_{L}C_{0}}$$

where *K_R_* is the dimensionless separation factor, and *C*_0_ is the initial concentration (mg/L). The parameter *K_R_* indicates the shape of the isotherm: *K_R_* > 1 is Unfavorable, *K_R_* = 1 is Linear, 0 < *K_R_* <1 is Favorable and *K_R_* = 0 is Irreversible.

Specific surface area occupied by Cu and Ag on biochars, *S* (m^2^/g), can be calculated using the following equation:

(9) $$S=\frac{Q_{m}LA}{M}$$

where *A* is the cross-sectional area of metal ion (m^2^) (for Cu: 1.58 Å^2^ and for Ag: 4.01 Å^2^), and *M* is the molecular weight of the metal (for Cu: 63.5 and for Ag: 107.9).

Efficiency, *E* (%), of Cu and Ag removal by biochars can be expressed as follows:

(10) $$E\%=\;\frac{C_{A}}{C_{0}}\cdot 100\%$$

where *C_A_* is the concentration of adsorbed ions (mg/L), and *C*_0_ is the initial concentration (mg/L).

## 4. Conclusions

The analysis of the results allowed the formulation of the following conclusions:

- Biochars obtained at 500 °C exhibited the highest specific surface areas but the lowest variable surface charges and the lowest contents of surface functional groups.
- Kinetics of the Cu and Ag sorption process were well-described by a pseudo-second-order model, and the IPD model revealed that Cu and Ag removals consisted of film diffusion, internal diffusion and mass action.
- Cu and Ag adsorptions on agricultural biochars were well-described by the Langmuir-Freundlich model.
- Agricultural biochar adsorption capacities of Cu and Ag increased with increasing the values of the pyrolysis temperature and with decreasing the valence of the metals.
- Monovalent Ag was better-removed than the divalent Cu ions from aqueous solutions. This was caused by the stronger attraction between the surface functional groups and Ag ions, which facilitated their penetration into biochar pores. This phenomenon was also dictated with the ionic radius of the adsorbates and electronegativity.
- Agricultural biochars produced at ≥500 °C demonstrate the highest removal efficiency of Cu and Ag and will have an important role as organic eco-adsorbents in the mono- and divalent heavy metal removals from aqueous media.

## Figures and Tables

**Table 1:** Characteristics of agricultural biochars with standard deviations (*n* = 3): SH3 (sunflower husks, 300 °C), SH4 (sunflower husks, 400 °C), SH5 (sunflower husks, 500 °C), SR3 (sunflower husks + rapeseed pomace, 300 °C), SR4 (sunflower husks + rapeseed pomace, 400 °C), SR5 (sunflower husks + rapeseed pomace, 500 °C), WW3 (wood waste, 300 °C), WW4 (wood waste, 400 °C) and WW5 (wood waste, 500 °C); *S_BET_*—specific surface area and *Q*—variable surface charge.

Type of Biochar	pH	*S_BET_* (m^2^/g)	*Q* (cmol/kg)	Carboxylic Groups (cmol/kg)	Lactonic Groups (cmol/kg)	Phenolic Groups (cmol/kg)	H/C	O/C
SH3	9.9 ± 3.0	71.7 ± 21.3	141.2 ± 11.1	30 ± 11	110 ± 22	190 ± 21	0.9 ± 0.01	0.4 ± 0.02
SH4	10.5 ± 2.0	78.8 ± 11.3	132.2 ± 10.6	30 ± 10	90 ± 11	190 ± 31	0.6 ± 0.02	0.2 ± 0.01
SH5	11.1 ± 2.0	85.6 ± 28.4	108.8 ± 11.2	20 ± 11	100 ± 31	130 ± 72	0.4 ± 0.01	0.2 ±0.01
SR3	10.1 ± 3.0	73.1 ± 23.4	281.6 ± 21.5	5 ± 2	185 ± 45	205 ± 52	1.0 ± 0.08	0.7 ± 0.04
SR4	11.2 ± 5.0	74.1 ± 23.3	237.1 ± 11.4	5 ± 2	135 ± 50	125 ± 81	0.8 ± 0.06	0.3 ± 0.08
SR5	11.3 ± 2.0	91.8 ± 18.4	203.6 ± 4.0	5 ± 1	105 ± 22	145 ± 75	0.4 ± 0.05	0.2 ± 0.01
WW3	8.1 ± 1.1	53.1 ± 41.1	127.9 ± 81.8	40 ± 10	130 ± 11	140 ± 24	0.7 ± 0.04	0.3 ± 0.01
WW4	9.5 ± 2.1	66.1 ± 3.4	87.2 ± 21.6	30 ± 11	140 ± 41	110 ± 52	0.6 ± 0.01	0.2 ± 0.02
WW5	10.1 ± 1.1	70.3 ± 14.2	96.2 ± 31.6	30 ± 11	110 ± 11	140 ± 20	0.4 ± 0.03	0.2 ± 0.01

**Table 2:** Parameters of the Cu adsorption kinetics calculated on the basis of the kinetic models with standard deviations (*n* = 3): SH3 (sunflower husks, 300 °C), SH4 (sunflower husks, 400 °C), SH5 (sunflower husks, 500 °C), SR3 (sunflower husks + rapeseed pomace, 300 °C), SR4 (sunflower husks + rapeseed pomace, 400 °C), SR5 (sunflower husks + rapeseed pomace, 500 °C), WW3 (wood waste, 300 °C), WW4 (wood waste, 400 °C) and WW5 (wood waste, 500 °C); *q_e_* is the heavy metal removal capacity at equilibrium; *k*_1_, *k*_2_ and *k_D_* are the reaction rate constants and *R*^2^ are the values of the correlation coefficients.

Cu	Pseudo-First-Order (Lagergren)			Pseudo-Second-Order (Ho and Mckay)			Intra-Particle Diffusion Model		
	*k*_1_ × 10^−2^ (1/min)	*q_e_* (mg/g)	*R* ^2^	*k*_2_ × 10^−2^ (g/mg·min)	*q_e_* (mg/g)	*R^2^*	*k_D_* × 10^−2^ (g/mg·min^1/2^)	*q_e_* (mg/g)	*R* ^2^
SH3	0.01 ± 0.01	6.0 ± 2.2	0.9 ± 0.4	4.5 ± 1.3	9.0 ± 1.1	0.99 ± 0.1	6.6 ± 1.9	5.7 ± 1.7	0.98 ± 0.1
SH4	0.09 ± 0.01	5.8 ± 2.2	0.8 ± 0.2	8.1 ± 1.8	9.2 ± 1.3	0.99 ± 0.1	6.6 ± 1.0	5.7 ± 1.7	0.98 ± 0.1
SH5	0.15 ± 0.01	5.9 ± 2.8	0.9 ± 0.3	8.2 ± 1.7	9.6 ± 0.5	0.99 ± 0.1	7.4 ± 1.7	5.7 ± 1.6	0.91 ± 0.2
SR3	0.11 ± 0.01	6.7 ± 2.7	0.9 ± 0.2	3.5 ± 1.0	9.0 ± 2.1	0.99 ± 0.1	8.2 ± 1.7	6.4 ± 1.2	0.94 ± 0.1
SR4	0.19 ± 0.01	7.8 ± 2.8	0.8 ± 0.1	7.3 ± 2.0	9.4 ± 1.1	0.99 ± 0.1	12.7 ± 2.9	6.8 ± 1.8	0.91 ± 0.3
SR5	0.35 ± 0.04	8.5 ± 2.6	0.9 ± 0.1	11.0 ± 1.7	9.6 ± 1.6	0.99 ± 0.1	13.5 ± 4.3	7.6 ± 1.5	0.93 ± 0.2
WW3	0.09 ± 0.01	4.6 ± 1.2	0.9 ± 0.1	1.1 ± 0.5	6.3 ± 1.3	0.99 ± 0.1	7.3 ± 1.4	4.2 ± 1.4	0.94 ± 0.1
WW4	0.12 ± 0.01	4.9 ± 1.0	0.8 ± 0.1	1.2 ± 2.7	6.3 ± 1.0	0.99 ± 0.1	8.8 ± 2.4	5.6 ± 1.4	0.93 ± 0.2
WW5	0.12 ± 0.01	5.0 ± 1.7	0.9 ± 0.3	1.9 ± 1.6	7.2 ± 2.0	0.99 ± 0.1	9.2 ± 4.5	7.6 ± 1.0	0.93 ± 0.1

**Table 3:** Parameters of the Ag adsorption kinetics calculated on the basis of the kinetic models with standard deviations (*n* = 3): SH3 (sunflower husks, 300 °C), SH4 (sunflower husks, 400 °C), SH5 (sunflower husks, 500 °C), SR3 (sunflower husks + rapeseed pomace, 300 °C), SR4 (sunflower husks + rapeseed pomace, 400 °C), SR5 (sunflower husks + rapeseed pomace, 500 °C), WW3 (wood waste, 300 °C), WW4 (wood waste, 400 °C) and WW5 (wood waste, 500 °C); *q_e_* is the heavy metal removal capacity at equilibrium; *k*_1_, *k*_2_ and *k_D_* are the reaction rate constants and *R*^2^ are the values of the correlation coefficients.

Ag	Pseudo-First-Order (Lagergren)			Pseudo-Second-Order (Ho and Mckay)			Intra-Particle Diffusion Model		
	*k*_1_ × 10^−2^ (1/min)	*q_e_* (mg/g)	*R* ^2^	*k*_2_ × 10^−2^ (g/mg·min)	*q_e_* (mg/g)	*R^2^*	*k_D_* × 10^−2^ (g/mg·min^1/2^)	*q_e_* (mg/g)	*R* ^2^
SH3	0.02 ± 0.01	9.1 ± 2.3	0.9 ± 0.3	4.2 ± 1.1	9.9 ± 1.0	0.99 ± 0.1	2.4 ± 0.2	7.3 ± 1.4	0.92 ± 0.1
SH4	0.02 ± 0.01	9.1 ± 3.1	0.9 ± 0.1	5.2 ± 1.5	10.0 ± 0.1	0.99 ± 0.1	4.6 ± 1.1	7.9 ± 1.4	0.96 ± 0.1
SH5	0.08 ± 0.01	9.1 ± 3.8	0.9 ± 0.1	6.1 ± 1.0	10.0 ± 0.4	0.98 ± 0.1	8.3 ± 2.8	8.7 ± 0.2	0.92 ± 0.1
SR3	0.04 ± 0.01	9.1 ± 3.7	0.9 ± 0.2	5.1 ± 0.9	9.1 ± 0.2	0.99 ± 0.1	10.1 ± 1.1	6.9 ± 3.7	0.97 ± 0.1
SR4	0.06 ± 0.01	9.1 ± 2.4	0.8 ± 0.1	7.9 ± 3.4	9.1 ± 0.6	0.99 ± 0.1	12.9 ± 2.4	7.7 ± 1.7	0.93 ± 0.1
SR5	0.14 ± 0.01	9.3 ± 1.0	0.9 ± 0.2	8.3 ± 2.8	10.1 ± 1.0	0.99 ± 0.1	14.7 ± 1.3	8.5 ± 1.7	0.93 ± 0.1
WW3	0.05 ± 0.01	5.2 ± 0.9	0.8 ± 0.1	1.5 ± 0.3	9.2 ± 0.3	0.99 ± 0.1	6.7 ± 1.8	4.3 ± 2.0	0.93 ± 0.1
WW4	0.05 ± 0.01	8.2 ± 2.1	0.9 ± 0.2	2.1 ± 0.2	10.0 ± 0.7	0.99 ± 0.1	8.4 ± 0.6	5.4 ± 1.9	0.94 ± 0.1
WW5	0.08 ± 0.01	9.0 ± 2.1	0.8 ± 0.3	2.2 ± 0.6	10.0 ± 0.8	0.99 ± 0.1	12.0 ± 1.7	6.1 ± 0.4	0.89 ± 0.1

**Table 4:** Isotherm parameters of the Cu adsorptions on the agricultural biochars with standard deviations (*n* = 3): SH3 (sunflower husks, 300 °C), SH4 (sunflower husks, 400 °C), SH5 (sunflower husks, 500 °C), SR3 (sunflower husks + rapeseed pomace, 300 °C), SR4 (sunflower husks + rapeseed pomace, 400 °C), SR5 (sunflower husks + rapeseed pomace, 500 °C), WW3 (wood waste, 300 °C), WW4 (wood waste, 400 °C) and WW5 (wood waste, 500 °C); *K_F_* and 1/*n* are the Freundlich constants, which represent the sorption capacity and the “heterogeneity parameter”; *K_L_* is the Langmuir constant related to the affinity of the adsorbate for the active sites; *Q_m_* is the maximum amount of metal ions in the monomolecular layer; *K_LF_* is related to the affinity of the adsorbate for the active sites; *A_m_* is the amount of available surface sites; *m* are the parameters determining the shape of the energy distribution function and *R*^2^ are the values of the correlation coefficients.

Cu	Freundlich Isotherm			Langmuir Isotherm			Langmuir-Freundlich Isotherm			
	*K_F_* (mg/g(L/mg)^1/*n*^)	*1/n*	*R^2^*	*K_L_* (L/mg)	*Q_m_* (mg/g)	*R* ^2^	*K_LF_* (L/mg)	*A_m_* (mg/g)	*m*	*R* ^2^
SH3	2.8 ± 0.5	0.3 ± 0.1	0.8 ± 0.5	0.2 ± 0.1	9.9 ± 2.1	0.92 ± 0.1	0.2 ± 0.1	6.4 ± 2.3	0.97 ± 0.2	0.99 ± 0.01
SH4	2.7 ± 0.7	0.3 ± 0.1	0.8 ± 0.5	0.3 ± 0.1	11.5 ± 3.5	0.95 ± 0.1	0.4 ± 0.1	7.3 ± 2.3	0.94 ± 0.1	0.98 ± 0.07
SH5	4.5 ± 1.5	0.2 ± 0.1	0.9 ± 0.1	0.5 ± 0.1	12.8 ± 5.2	0.94 ± 0.1	0.4 ± 0.1	9.3 ± 4.1	0.89 ± 0.2	0.99 ± 0.05
SR3	0.9 ± 0.2	0.7 ± 0.1	0.8 ± 0.2	0.2 ± 0.1	14.3 ± 4.3	0.94 ± 0.1	0.5 ± 0.1	3.0 ± 1.1	0.98 ± 0.2	0.98 ± 0.04
SR4	4.1 ± 1.1	0.4 ± 0.1	0.8 ± 0.1	0.4 ± 0.2	14.9 ± 4.0	0.95 ± 0.1	0.5 ± 0.1	10.6 ± 4.2	0.89 ± 0.1	0.99 ± 0.05
SR5	5.4 ± 1.0	0.4 ± 0.1	0.9 ± 0.1	0.5 ± 0.1	16.2 ± 5.1	0.93 ± 0.1	1.3 ± 0.2	11.5 ± 6.7	0.88 ± 0.1	0.98 ± 0.01
WW3	0.5 ± 0.1	0.4 ± 0.2	0.9 ± 0.1	0.1 ± 0.1	1.8 ± 2.0	0.95 ± 0.05	0.3 ± 0.1	0.6 ± 2.1	0.99 ± 0.1	0.99 ± 0.04
WW4	0.5 ± 0.1	0.4 ± 0.3	0.9 ± 0.1	0.3 ± 0.1	3.5 ± 3.1	0.95 ± 0.07	0.5 ± 0.1	1.1 ± 2.9	0.95 ± 0.3	0.99 ± 0.03
WW5	1.4 ± 0.2	0.3 ± 0.1	0.8 ± 0.2	0.3 ± 0.1	3.5 ± 2.5	0.94 ± 0.05	1.0 ± 0.1	1.8 ± 1.1	0.94 ± 0.2	0.99 ± 0.09

**Table 5:** Isotherm parameters of the Ag adsorptions on the agricultural biochars with standard deviations (*n* = 3): SH3 (sunflower husks, 300 °C), SH4 (sunflower husks, 400 °C), SH5 (sunflower husks, 500 °C), SR3 (sunflower husks + rapeseed pomace, 300 °C), SR4 (sunflower husks + rapeseed pomace, 400 °C), SR5 (sunflower husks + rapeseed pomace, 500 °C), WW3 (wood waste, 300 °C), WW4 (wood waste, 400 °C) and WW5 (wood waste, 500 °C); *K_F_* and 1/*n* are the Freundlich constants, which represent the sorption capacity and the “heterogeneity parameter”; *K_L_* is the Langmuir constant related to the affinity of the adsorbate for the active sites; *Q_m_* is the maximum amount of metal ions in the monomolecular layer; *K_LF_* is related to the affinity of the adsorbate for the active sites; *A_m_* is the amount of available surface sites; *m* are the parameters determining the shape of the energy distribution function and *R*^2^ are the values of the correlation coefficients.

Ag	Freundlich Isotherm			Langmuir Isotherm			Langmuir-Freundlich Isotherm			
	*K_F_* (mg/g(L/mg)^1/*n*^)	*1/n*	*R^2^*	*K_L_* (L/mg)	*Q_m_* (mg/g)	*R* ^2^	*K_LF_* (L/mg)	*A_m_* (mg/g)	*m*	*R* ^2^
SH3	7.9 ± 1.2	0.6 ± 0.1	0.84 ± 0.3	0.2 ± 0.1	10.9 ± 2.9	0.94 ± 0.04	0.2 ± 0.1	14.5 ± 2.2	0.99 ± 0.1	0.99 ± 0.04
SH4	8.1 ± 1.5	0.6 ± 0.1	0.84 ± 0.2	0.3 ± 0.1	14.1 ± 2.4	0.94 ± 0.03	0.8 ± 0.2	18.3 ± 2.2	0.97 ± 0.1	0.98 ± 0.03
SH5	9.0 ± 1.6	0.4 ± 0.1	0.78 ± 0.2	0.4 ± 0.1	22.3 ± 2.4	0.91 ± 0.04	1.2 ± 0.6	21.7 ± 1.8	0.96 ± 0.1	0.99 ± 0.06
SR3	8.4 ± 1.1	0.4 ± 0.1	0.77 ± 0.1	0.3 ± 0.1	6.9 ± 3.17	0.93 ± 0.04	0.8 ± 0.2	16.1 ± 4.5	0.88 ± 0.1	0.99 ± 0.05
SR4	9.9 ± 1.7	0.3 ± 0.1	0.71 ± 0.1	0.3 ± 0.1	10.6 ± 2.8	0.94 ± 0.02	0.9 ± 0.1	27.9 ± 6.8	0.74 ± 0.1	0.99 ± 0.03
SR5	11.0 ± 1.1	0.3 ± 0.01	0.76 ± 0.3	0.5 ± 0.1	14.0 ± 5.1	0.93 ± 0.04	1.6 ± 0.3	46.2 ± 2.3	0.62 ± 0.1	0.99 ± 0.04
WW3	3.7 ± 1.4	0.5 ± 0.2	0.77 ± 0.1	0.5 ± 0.1	9.6 ± 1.2	0.92 ± 0.06	0.4 ± 0.1	12.1 ± 1.0	0.98 ± 0.1	0.98 ± 0.04
WW4	5.3 ± 1.6	0.4 ± 0.1	0.84 ± 0.5	0.6 ± 0.2	14.6 ± 2.5	0.90 ± 0.01	0.3 ± 0.1	19.2 ± 3.5	0.83 ± 0.1	0.99 ± 0.05
WW5	5.7 ± 1.0	0.4 ± 0.1	0.79 ± 0.7	0.7 ± 0.1	17.8 ± 2.2	0.90 ± 0.03	1.0 ± 0.2	26.8 ± 4.9	0.50 ± 0.1	0.99 ± 0.01

**Table 6:** Specific surface areas occupied by the metal ions (m^2^/g) with standard deviations (*n* = 3): SH3 (sunflower husks, 300 °C), SH4 (sunflower husks, 400 C), SH5 (sunflower husks, 500 °C), SR3 (sunflower husks + rapeseed pomace, 300 °C), SR4 (sunflower husks + rapeseed pomace, 400 °C), SR5 (sunflower husks + rapeseed pomace, 500 °C), WW3 (wood waste, 300 °C), WW4 (wood waste, 400 °C) and WW5 (wood waste, 500 °C).

	SH3	SH4	SH5	SR3	SR4	SR5	WW3	WW4	WW5
Cu	1.5 ± 0.3	1.7 ± 0.8	1.9 ± 0.2	1.2 ± 0.3	1.7 ± 0.4	2.4 ± 0.3	0.3 ± 0.1	0.6 ± 0.2	0.6 ± 0.1
Ag	2.7 ± 1.2	3.4 ± 0.5	4.2 ± 0.4	3.8 ± 1.1	4.6 ± 1.8	5.6 ± 1.8	2.2 ± 0.9	3.3 ± 1.1	4.0 ± 1.2

**Table 7:** Biochar efficiency (%) with standard deviations (*n* = 3) for 300 mg/L: SH3 (sunflower husks, 300 °C), SH4 (sunflower husks, 400 °C), SH5 (sunflower husks, 500 °C), SR3 (sunflower husks + rapeseed pomace, 300 °C), SR4 (sunflower husks + rapeseed pomace, 400 °C), SR5 (sunflower husks + rapeseed pomace, 500 °C), WW3 (wood waste, 300 °C), WW4 (wood waste, 400 °C) and WW5 (wood waste, 500 °C).

	SH3	SH4	SH5	SR3	SR4	SR5	WW3	WW4	WW5
Cu	40.7 ± 12.3	43.3 ± 7.4	44.2 ± 21.4	69.2 ± 3.6	71.4 ± 12.2	71.5 ± 25.6	13.7 ± 14.7	19.1 ± 2.3	20.3 ± 7.8
Ag	93.6 ± 24.0	98.5 ± 9.9	99.2 ± 13.9	77.2 ± 18.7	95.6 ± 12.1	99.4 ± 17.9	43.4 ± 24.9	50.3 ± 25.9	65.4 ± 16.3
